# Giant hydronephrosis management in the Era of minimally invasive surgery: A case series

**DOI:** 10.1016/j.ijscr.2020.09.144

**Published:** 2020-09-23

**Authors:** Abdulrahman Alsunbul, Tarek Alzahrani, Abdulrahman Binjawhar, Abdullah Aldughiman, Hossam S. El-Tholoth, Ahmed Alzahrani, Hamad Alakrash

**Affiliations:** Prince Sultan Military Medical City, Urology Department, Riyadh, Saudi Arabia

**Keywords:** GH, giant hydronephrosis, UPJO, ureteropelvic junction obstruction, Giant hydronephrosis, Nephrectomy, Nephrostomy, Laparoscopy

## Abstract

**Introduction:**

Giant hydronephrosis (GH) is a rare urological entity, described as more than 1 L of fluid contained in the renal collecting system. Ureteropelvic junction obstruction (UPJO) is the most common cause. GH if not discovered and managed early can result in long term complications. We present our experience in the late presentation of adult Giant hydornephrosis.

**Presentation of cases:**

We reviewed all the cases of patients with giant hydronephrosis who presented to our institute from December 2017–December 2019 at our institute. Pre-operative renal ultrasound, computed tomography with contrast and MAG-3 were performed on all patients to establish their diagnoses. The patients' demographic data, clinical presentation, preparatory investigations, indications for intervention, type of intervention, pre- and post-operative complications and durations of hospital stay were reported. Laparoscopic transperitoneal nephrectomy was completed in three cases without open conversion. One case proceeded to open conversion owing to a lack of space and severe adhesions. The mean operating time was 79.7 min (range: 65–95 min), estimated blood loss was 75 mL and the mean hospital stay was 4 days (range: 2–6 days).

**Discussion:**

The first case of GH was described in 1746. Since then, few cases have been described in the literature. A radiological definition, is the occupation of the hemi-abdomen by the kidney with a midline cross which is the height of five vertebral bodies. GH may be congenital or acquired. The most common presentation of GH is abdominal distention followed by fever and flank pain.

**Conclusion:**

When nephrectomy is indicated in giant hydronephrosis, the laparoscopic trans-peritoneal approach is feasible. Pre-operative decompression using a nephrostomy tube and suspension stitch use facilitate the surgery.

## Highlights

• We reported on the laparoscopic trans-peritoneal approach for giant hydronephrosis.
• No major peri-operative complications were reported.
• Pre-operative decompression using a nephrostomy tube facilitates the surgery.

## 1 Introduction

Giant hydronephrosis (GH) is a rare urological entity, described in the literature as more than 1 L of fluid contained in the renal collecting system [1]. Its presentation is usually vague, but it is most frequently associated with abdominal distention. The most common cause of the condition, described in the literature, is ureteropelvic junction obstruction (UPJO) [1]. A limited number of cases of adult GH has been reported. GH if not discovered and managed early can result in long term complications such as hypertension, renal failure, renal rupture, and loss of renal unit [2]. here we present our experience in the late presentation of adult Giant hydornephrosis. The work reported herein adheres to the PROCESS criteria [3].

## 2 Presentation of cases

**Fig. 1:**
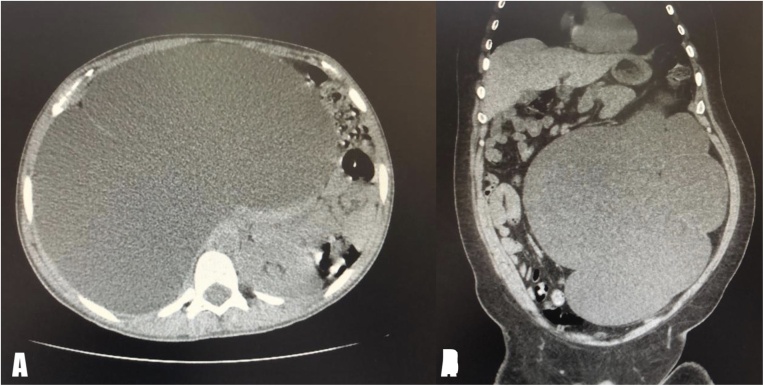
(A, B): Axial and coronal CT scan showed severe left hydronephrosis crossing the midline with thinning of renal parenchyma.

After institutional ethical committee clearance was taken for the project. A retrospective review of all cases of nephrectomies performed between December 2017–December 2019 at our institute. Among them, four patients presented with massive hydronephrosis (GH). The main complaint of all four patients (two male, two female) was flank pain, with two patients experiencing more pain on the right side and the other two with more pain on the left side. Clinical examination revealed abdominal distention in all cases, with costophrenic angle tenderness in two cases. Renal profiles were normal in all the cases. All patients underwent pre-operative sonography, CT scan (Fig. 1), and nuclear renal scan which showed a renal split function of <15% in all cases, with preserved anatomy and functioning on the contralateral side. Two patients required gradual decompression by nephrostomy tube insertion pre-surgery in the affected kidney due to presence of urinary tract infection, both patients were observed for any signs of acute kidney injury and cardiopulmonary distress post drainage. The patients’ characteristics are displayed in (Table 1).

**Table 1:** This table summarizes patients age, laterality, preoperative renal scan result, pre-operative drainage, estimated blood loss, hospital stay, and operative time.

PATIENT	AGE (years)	WEIGHT (kg)	INDICATION	LATERALITY	PREOPERATIVE RENAL FUNCTION ON RENAL SCAN	PRE-OPERATIVE DRAINGE	BLOOD LOSS (ml)	HOSPITAL STAY (days)	OPERATIVE TIME (min)
#1	29	70	FLANK PAIN	LEFT	14%	NON	30	2	95
#2	26	82	FLANK PAIN	RIGHT	COMPLETE NEPHRON FUNCTION LOSS	NON	20	3	90
#3	48	86	FLANK PAIN	LEFT	10%	YES	25	6	65
#4	22	65	FLANK PAIN + RECURRENT UTI	RIGHT	COMPLETE NEPHRON FUNCTION LOSS	YES	10	5	69

**Fig. 2:**
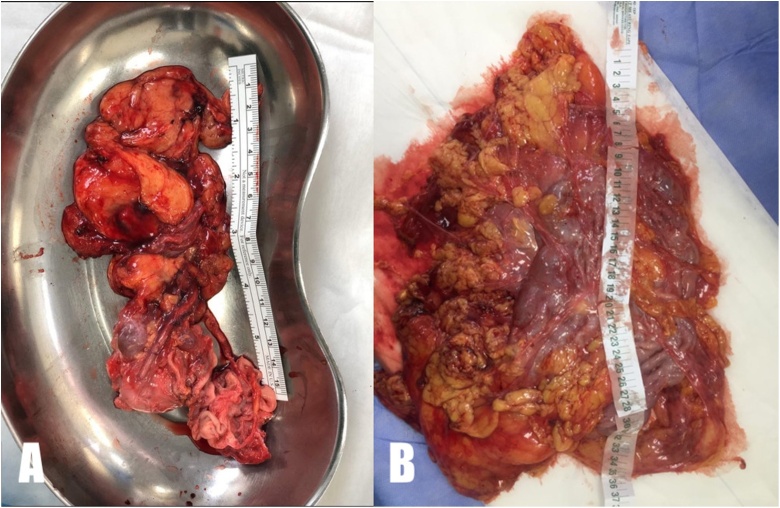
(A, B): Gross appearance of kidney with giant hydronephrosis after laparoscopic nephrectomy in two different patients.

Surgery was performed laparoscopically using a trans-peritoneal approach (Fig. 2); patients were position in a 30° flank-up position.

Pneumoperitoneum was achieved through the open Hasson technique. (Four ports were used: a 5-mm subcostal port in the anterior axillary line; a 10-mm camera port halfway between the subcostal port and the umbilicus, and a 12-mm port 2 cm supero-medial to the anterior iliac supine, which is used for instrumentation and the passage of sutures or staplers to secure and divide hilar vessels. In all cases, a suspension stitch in the renal pelvis, through the anterior abdominal wall, was used to assist retraction for better identification of the renal hilum. Upper and lower windows were achieved after releasing the adhesions present in all cases. The renal hilum was controlled en bloc using a 60-mm vacular endostapler loaded with 2.5-m titanium clips.

In patient #4 (Table 1), laparoscopic surgery was converted to an open procedure due to lack of space and severe adhesions surrounding the kidney. A crossing vessel was observed in one patient intra-operatively. The mean operating time was 79.7 min (range: 65−95 min), estimated blood loss was 75 mL and the mean hospital stay was 4 days (range: 2–6 days). None of the patients required drains intra-operatively and there were no major peri-operative complications. Patients were discharge in a good health status with follow up in the clinic. follow up clinical examination showed no sign of hypertension in all cases. Renal Ultrasonography was unremarkable for all patients along with normal renal function test.

## 3 Discussion

The first case of GH was described in 1746. Since then, few cases have been described in the literature [1]. This condition is a rare urological entity, defined in the literature as more than 1 L of fluid or fluid amounting to at least 1.6% of one’s body weight, contained in the collecting system. A radiological definition, first described by Crooks et al. [4], is the occupation of the hemi-abdomen by the kidney with a midline cross which is the height of five vertebral bodies [2]. GH is more commonly reported in children than in adults. GH may be congenital or acquired but is mostly congenital and commonly caused by UPJO. Less commonly, ureteral ectopia, a duplicated collecting system, and aberrant vasculature causing extrinsic compression of the UPJO may produce GH. Acquired causes include ureteral calculus, trauma, ischemia, carcinoma, and retroperitoneal fibrosis [5]. Our cases presented with progressive abdominal distention, and two of them were associated with urinary tract infection which required urgent decompression. The final diagnosis was UPJO for all the cases. The most common presentation of GH is abdominal distention followed by fever, flank pain, hematuria, acute abdominal pain and less commonly, recurrent urinary tract infections [2].

With the right kidney mostly affected, the mean amount of fluid drained immediately on nephrostomy tube placement was 3.5 ± 0.6 L in adults and 1.9 ± 0.4 L in children. This significant difference can be explained by the delayed presentation in adults, who have a higher capacity of the retroperitoneal space to accommodate excess fluid, compared to children [2]. Complications of GH include renal failure, hypertension and mechanical obstruction to the adjacent organs. One of the known complications post nephrostomy insertion, is acute renal injury and cardiopulmonary distress, where the latter may be due to rapid decompression of the hydronephrotic kidney, which may then lead to a change in the hemodynamic balance. This is known as paracentesis induced circulatory dysfunction. This usually occurs after draining large volumes. The theory behind this is that with large amounts of intra-abdominal fluid, filling of the right atrium tends to be incomplete; as fluid is drained, venous return to the right atrium increases with a resultant increase in cardiac output and splanchnic vasodilation, leading to a severe decrease in the mean arterial pressure [2]. Diagnosis is usually performed with ultrasound, CT, and MAG 3, and treatment will depend on the diagnostic findings. The decision between nephrectomy and kidney sparing therapy is critical, and most cases described in the literature are treated either by simple nephrectomy or renal sparing surgery. Some studies report the rate of nephrectomy as being up to 30% and others report a rate of up to 70% [4,6]. Most of the reported cases are managed by open nephrectomy and gradual decompression by nephrostomy tube [7]. The management of GH should be treated on a case-by-case basis depending on salvageability of the affected kidney and accessibility of kidney-sparing therapy.

We are aware about the limitations of our study mainly the sample number was small and the retrospective design.

## 4 Conclusion

The management of GH should be treated on a case-by-case basis depending on salvageability of the affected kidney and accessibility of kidney-sparing therapy. When nephrectomy is indicated in giant hydronephrosis, the laparoscopic trans-peritoneal approach is feasible, with pre-operative decompression by nephrostomy tube and the application of a suspension stitch to facilitate the surgery.

## Declaration of Competing Interest

The authors report no declarations of interest.

## Source of funding

This research did not receive any specific grant from funding agencies in the public.

## Ethical approval

Preliminary ethical approval, to be received with reference number during this week.

## Consent

Patients have given their consents for the study to be published. A copy of the written consent is available, at any time.

## Author contribution

Abdulrahman Alsunbul: final manuscript.

Tarek Alzahrani: paraphrasing.

Abdulrahman Binjawhar: draft of the manuscript and review of the literature.

Abdullah Aldughiman: draft of the manuscript and review of the literature.

Hossam S El-Tholoth: review of the literature.

Ahmed Alzahrani: review of the literature.

Hamad Alakrash: primary consultant & Surgeon final modification.

## Registration of research studies

1. Name of the registry: Research registry.
2. Unique identifying number or registration ID: researchregistry5817.
3. Hyperlink to your specific registration (must be publicly accessible and will be checked): https://www.researchregistry.com/browse-the-registry#home/registrationdetails/5f118c16d34e620015e87c8e/.

## Guarantor

Abdulrahman Alsunbul.

## Provenance and peer review

Not commissioned, externally peer-reviewed.
